# Effectiveness and tolerability of givosiran for the management of acute hepatic porphyria: A monocenter real-life evaluation

**DOI:** 10.1016/j.ymgmr.2024.101111

**Published:** 2024-06-22

**Authors:** Claudio Carmine Guida, Maria Nardella, Aurora del Mar YS Perez, Maria Savino, Gaetano Ferrara, Francesco Napolitano, Annalisa Crisetti, Francesco Aucella, Filippo Aucella

**Affiliations:** Scientific Institut for Research and Health Care, Fondazione Casa Sollievo della Sofferenza, 71013 San Giovanni Rotondo, Italy

**Keywords:** Acute hepatic porphyria, Acute intermittent porphyria, Givosiran, RNA interference, Quality of life

## Abstract

Acute hepatic porphyrias (AHPs) are a family of rare, autosomal, dominantly inherited conditions characterized by abnormalities in the production of heme. Advances in molecular engineering have provided new therapeutic possibilities for modifying the heme synthetic pathway in patients with porphyria. In particular, the RNA interference therapeutic givosiran was approved for the treatment of adults and adolescents with AHP aged >12 years based on the positive results of the phase III trial ENVISION. Despite the extended characterization of the activity of givosiran in clinical trials, reports on the long-term effects and effectiveness of the treatment in clinical practice are still scant. To fill this gap, this case series describes a monocentric Italian cohort of AHP patients treated with givosiran. Overall, our real-life experience supports the clinical evidence that long-term treatment with givosiran is well tolerated and able to provide sustained and continuous benefit to patients with acute intermittent porphyria, as reflected by the reduction in the frequency of attacks. In our series, givosiran treatment was also associated with improvement in assessments of quality of life, pain and fatigue.

## 1 Introduction

Acute hepatic porphyrias (AHPs) are a family of rare, autosomal, dominantly inherited conditions characterized by abnormalities in the production of heme, a crucial component of hemoglobin and other hemoproteins [5,12]. AHPs include acute intermittent porphyria (AIP), variegate porphyria (VP), hereditary coproporphyria (HCP), and 5-aminolevulinic acid (ALA) dehydratase deficiency porphyria (ADP). Clinically, AHPs present with severe acute symptoms such as abdominal pain, arm/leg pain and back pain, in addition to chronic symptoms such as nausea, vomiting, constipation, muscle weakness, neuropathy, tachycardia and hypertension, change in mental status [5,21].

AIP represents the most common form among AHPs [5]. It is caused by a deficiency of the hydroxymethylbilane synthase (HMBS) enzyme, which leads to the accumulation of porphyrins and their precursors, delta-aminolevulinic acid (ALA) and porphobilinogen (PBG), particularly in the liver, bone marrow and nervous system [7,8].

Symptoms of AIP typically appear during adolescence or early adulthood [3]. In addition to the common symptoms of AHPs mentioned above, other AIP symptoms may include tingling or numbness in the limbs, seizures, confusion, hallucinations, anxiety and depression. AIP can also affect the autonomic nervous system, leading to high blood pressure, rapid heart rate, and urinary retention [8,23]. Various factors, such as hormonal changes, medications, diet, and environmental triggers, can precipitate an acute attack in susceptible individuals. At the same time, chronic symptoms were frequently reported, with daily manifestations that lead to impaired quality of life due to comorbidities, concomitant medications and increased healthcare utilization [5,20]. For instance, chronic symptoms of the disease may present even without a history of acute attacks [13].

Diagnosis of AHPs is based on a combination of clinical and biochemical evaluations [6]. Measurement of porphyrin levels in the urine, blood or stool can help detect the accumulation of porphyrins and their precursors, and genetic testing can further confirm the presence of the mutated gene responsible for AIP [4]. For instance, not all individuals with the *HMBS* gene mutation will experience symptoms, and, at the same time, identification of the *HMBS* mutation is not reported in all diagnosed patients [2]. Accordingly, literature evidence report that genetic analysis does not identify mutations in all unequivocally diagnosed cases and, therefore, cannot be used to exclude a diagnosis of porphyria [22].

Traditionally, treatment of AHPs primarily focused on preventing and managing acute attacks with supportive care, avoiding trigger factors able to induce heme synthesis, such as certain medications, alcohol and fasting [3]. During an acute attack, symptomatic treatment may include intravenous administration of glucose and/or heme preparations to suppress heme synthesis and reduce porphyrin production [3]. Due to the absence of alternative therapeutic modalities, patients with recurrent attacks were often managed by prophylactic heme therapy, even if off-label, with good results [1]. However, the long-term side effects of heme prophylactic therapy included dependence on the treatment, thrombotic complications and secondary hemochromatosis [5,10]. In addition, patients in prophylactic treatment with heme reported a diminished quality of life (QoL), increased healthcare utilization and a significant economic burden of the disease [5].

Advances in molecular engineering have provided new therapeutic possibilities for modifying the heme synthetic pathway in patients with porphyria. In particular, the RNA interference (RNAi) therapeutic givosiran (GIVLAARI®, Alnylam Pharmaceuticals, Cambridge, MA, USA) was recently designed to target the aminolevulinic acid synthase 1 (ALAS1) enzyme to inhibit the production of ALA [14]. The use of givosiran was approved for the treatment of adults and adolescents with AHP aged >12 years based on the positive results of the phase III trial ENVISION [2,9,17,19]. The study included 94 recurrent AHP patients with a genetic diagnosis in 95% of cases [2,9,19]. The majority of included patients had AIP (95%) [2]. In the study, givosiran was able to lower significantly the rate of acute attacks and reduce the levels of urinary ALA and PBG compared with the placebo group [2,9,19]. Reported key adverse events were related to liver and kidney function, with reported increases in serum aminotransferase levels and the estimated glomerular filtration rate (GFR) in givosiran-treated participants [2,9,19].

Despite the extended characterization of the activity of givosiran in clinical trials [15,19], reports on the long-term effects and effectiveness of the treatment in clinical practice are still scant [[11], [18]]. To fill this gap, this case series describes a monocentric Italian cohort of AHP patients treated with givosiran. We reported data on treatment effectiveness, with a focus on kidney and liver function, as well as on safety and QoL.

## 2 Patients and methods

A monocentric, retrospective study was conducted at the Scientific Institute “Casa Sollievo della Sofferenza” (San Giovanni Rotondo, Foggia, Italy) to collect clinical and biological data of patients diagnosed with AIP in treatment with givosiran starting in 2020. Adult (>18 years) patients in treatment with givosiran based on the physician's judgment, according to the SmPC, were considered. Due to the retrospective nature of the case series, treatment regimens and patient education were not standardized.

The study was conducted in accordance with the ethical principles of the revised version of the Declaration of Helsinki (52nd WMA General Assembly, Edinburgh, Scotland, October 2000). All patients provided written informed consent to treatment and the publication of clinical data.

### 2.1 Study measures

#### 2.1.1 Effectiveness

The effectiveness parameters (ALA, PBG) and parameters related to renal function (serum creatinine, GFR) were retrieved from medical records at baseline, after 6 and 12 months of treatment (ALA, PBG) and at the last control. Effectiveness was assessed by comparing baseline values with the last available value in the study period. For instance, with regard to our center, the blood chemistry values are checked on a monthly basis. In case of acute crisis, patients who enter the hospital undergo urinary ALA and PBG and peak plasma porphyrins during the crisis. The mean annualized rate of attacks was also assessed before and after the start of the treatment.

#### 2.1.2 Safety

Parameters related to liver functions (glutamic-oxaloacetic transaminase, glutamic-pyruvic transaminase) were reported at the baseline and at the last control to assess treatment safety.

#### 2.1.3 Quality of life

Quality of life was assessed through the SF-12v1 Italian version questionnaire, brief pain inventory (BPI) and brief fatigue inventory (BFI) questionnaires. The SF-12v1 Italian version questionnaire was used to measure the perception of health-related quality of life. The SF-12 has two dimensions: the physical composite (PCS) and the mental composite (MCS) scores. PCS and MCS scores range from 0 to 100, with higher scores indicating better functioning. The BPI questionnaire was used to measure both the intensity of the pain (sensory dimension-PIS) and the interference of pain in the patient's life (reactive dimension-API). For both, 0 = no interference/pain and 10 = interferes completely/pain is as bad as you can imagine. The BFI questionnaire was also used to quickly assess the severity of fatigue experienced by patients. A 0–90 score scale is used; higher scores correspond to more severe fatigue. Only patients with QoL questionnaires available at baseline, 6 and 12 months were considered.

### 2.2 Statistical analysis

Descriptive statistics were used to assess effectiveness and safety. The Shapiro–Wilk test was performed to evaluate the normal distribution of the data related to the QoL assessment. Median, first and third quartiles were reported as summary and variability measures, respectively, and the violin plot was used as a visualization method.

## 3 Case series

A total of 11 patients with AIP were included, predominantly females (*n* = 10; 90%) (Table 1). A genetic diagnosis was available for 7 (63%) patients. The mean age at disease onset was 37 years old (SD, standard deviation: 8 years). Each patient was treated with monthly subcutaneous injections of 2.5 mg/kg givosiran. The mean duration of follow-up under treatment was 16 months (SD: 5 months). All patients were treated with glucose and/or heme preparations to manage acute attacks (3 mg/kg/day for 4 consecutive days). Seven patients (63%) were undergoing prophylactic heme before treatment with givosiran; the mean time of exposure to heme was 4 years (SD: 1 year). Prophylactic heme was administered monthly via a central venous catheter to all patients, requiring hospital access and concomitant administration of acetylsalicylic acid to prevent catheter-related thrombosis. All patients had homocysteine values within the normal range and were treated with prophylactic folic acid in nine out of 11 cases (81%). Table 1 summarizes the main clinical, genetic and biochemical characteristics of patients at the baseline.

**Table 1:** Clinical and genetic characteristics of patients.

ID	Sex	Age (years)	AHP type	Age at diagnosis (years)	Mutation	Prophylactic heme before treatment	Exposure time to heme (years)	Time from treatment initiation (months)	Treatment with folic acid (up to April 2023)
1	Female	37	AIP	32	c.874C > T	Yes	3	18	Yes
2	Female	53	AIP	33	c.652-2delA	Yes	6	22	Yes
3	Female	58	AIP	46	c.652-2delA	Yes	6	22	Yes
4	Male	36	AIP	26	No	Yes	5	22	Yes
5	Female	35	AIP	34	c.874C > T	No	–	11	Yes
6	Female	35	AIP	33	c.874C > T	No	–	18	Yes
7	Female	54	AIP	53	c.181delG	No	–	8	No
8	Female	48	AIP	42	c.652-2delA	Yes	2	21	Yes
9	Female	55	AIP	42	c.613-31 A > G	Yes	5	17	Yes
10	Female	52	AIP	45	No	Yes	4	17	Yes
11	Female	26	AIP	25	No	No	–	6	No

### 3.1 Treatment effectiveness

The mean (±SD) annualized attack rate before treatment was 7 ± 3. From the start of givosiran treatment and up to the time of the study, every patient was free of acute crisis.

Givosiran treatment resulted in a decrease in urinary ALA levels in nine patients (82%) and PBG urinary levels in all patients (Table 2). The mean percentage reduction in urinary ALA from baseline was 71.6% (SD: 21.4%). Nine patients (82%) had an ALA level under 5 μmol/mmol creatinine. Two patients showed a moderate increase in ALA levels, although always under the level of 5 μmol/mmol creatinine. The mean percent reduction in urinary PBG from baseline was 75.0% (SD: 21.7%) (Table 2).

**Table 2:** Effectiveness parameters.

ID	ALA*					PBG**					Time from treatment initiation (months)
	Baseline (μmol/mmol creatinine)	After 6 months (μmol/mmol creatinine)	After 12 months (μmol/mmol creatinine)	Most recent follow-up(μmol/mmol creatinine)	Reduction from baseline (%)	Baseline (μmol/mmol creatinine)	After 6 months (μmol/mmol creatinine)	After 12 months (μmol/mmol creatinine)	Most recent follow-up (μmol/mmol creatinine)	Reduction from baseline (%)	
1	8.57	0.93	1.59	1.23	−86.0	37.61	1.74	5.25	3.13	−91.7	18
2	16.20	1.59	1.81	1.56	−91.0	73.56	4.29	3.00	2.70	−96.4	22
3	16.12	4.53	1.61	1.38	−91.5	29.93	4.03	1.06	1.52	−95.0	22
4	7.51^a^	2.21	2.29	2.52	−66.7	2.18^a^	0.75	0.94	0.63	−71.2	22
5	1.2	5.20	1.60	N/A	+33.0	2.35	2.38	0.64	N/A	−72.2	11
6	5.25	0.58	1.14	1.39	−75.0	32.46	0.51	2.66	3.27	−90.0	18
7	2.45	1.31	N/A	N/A	−53.4	1.21	1.02	N/A	N/A	N/A	8
8	12.6	6.09	0.56	3.69	−29.2	2.41	2.39	0.77	1.30	−46.1	21
9	2.52	2.53	1.98	3.49	+ 38.0	9.30	2.89	1.97	2.26	−77.2	17
10	1.07^b^	0.52	2.36	4.18	N/A	0.64^b^	0.7	1.34	1.23	N/A	17
11	6.46^c^	1.29	N/A	N/A	−80.0	6.26^c^	2.19	N/A	N/A	−35.3	6

ALA: delta-aminolevulinic acid; PBG: porphobilinogen.

* ALA reference range: upper limit of normal, 1.47 mmol/mol creatinine; ** PBG reference range: upper limit of normal, 0.14 mmol/mol creatinine.

a ALA max value: 8.41 μmol/mmol creatinine; PBG max value: 2.18 μmol/mmol creatinine.

b ALA max value: 10.56 μmol/mmol creatinine; PBG max value: 3.67 μmol/mmol creatinine.

c ALA max value: 6.46 μmol/mmol creatinine; PBG max value: 6.26 μmol/mmol creatinine.

No patient was hyponatremic at baseline nor experienced hyponatremia during treatment. Serum creatinine was in the normal range (0.5–1.2 mg/dL) during treatment in all patients, as well as GFR (Table 3).

**Table 3:** Kidney function parameters.

ID	Serum creatinine*		GFR CKD**		Time from treatment initiation (months)
	Baseline (mg/dL)	Most recent follow-up (mg/dL)	Baseline (mg/dL)	Most recent follow-up (mg/dL)	
1	1.6	1.5	N/A	44	18
2	1.1	1.3	58	47	22
3	0.9	0.9	71	71	22
4	0.7	0.9	122	101	22
5	0.5	0.5	127	127	11
6	1.4	1.2	49	59	18
7	0.9	1.0	73	64	8
8	0.5	0.7	116	104	21
9	0.5	0.6	111	104	17
10	0.8	0.9	85	74	17
11	0.5	0.4	135	145	6

GFR CKD: Creatinine equation for glomerular filtration rate.

*Serum creatinin normal range: 0.5–1.2 mg/dL; ** GFR CKD EPI creatinine equation.

### 3.2 Safety

Liver function parameters (glutamic-oxaloacetic transaminase, glutamic-pyruvic transaminase) remained within the normal range during treatment (Table 4). No patients discontinued the treatment.

**Table 4:** Liver function parameters.

ID	GOT*		GPT**		Time from treatment initiation (months)
	Baseline (U/l)	Most recent follow-up (U/l)	Baseline (U/l)	Most recent follow-up (U/l)	
1	26	23	31	25	18
2	26	20	24	21	22
3	28	37	30	38	22
4	17	41	24	70	22
5	18	19	27	20	11
6	41	30	46	41	18
7	14	29	26	43	8
8	17	29	N/A	24	21
9	28	29	45	26	17
10	N/A	30	25	32	17
11	23	31	33	51	6

GOT: glutamic-oxaloacetic transaminase; GPT: glutamic-pyruvate transaminase.

*GOT normal ranges values: 9–40 U/l; ** GPT normal range values: 7–57 U/l.

### 3.3 Quality of life

**Fig. 1:**
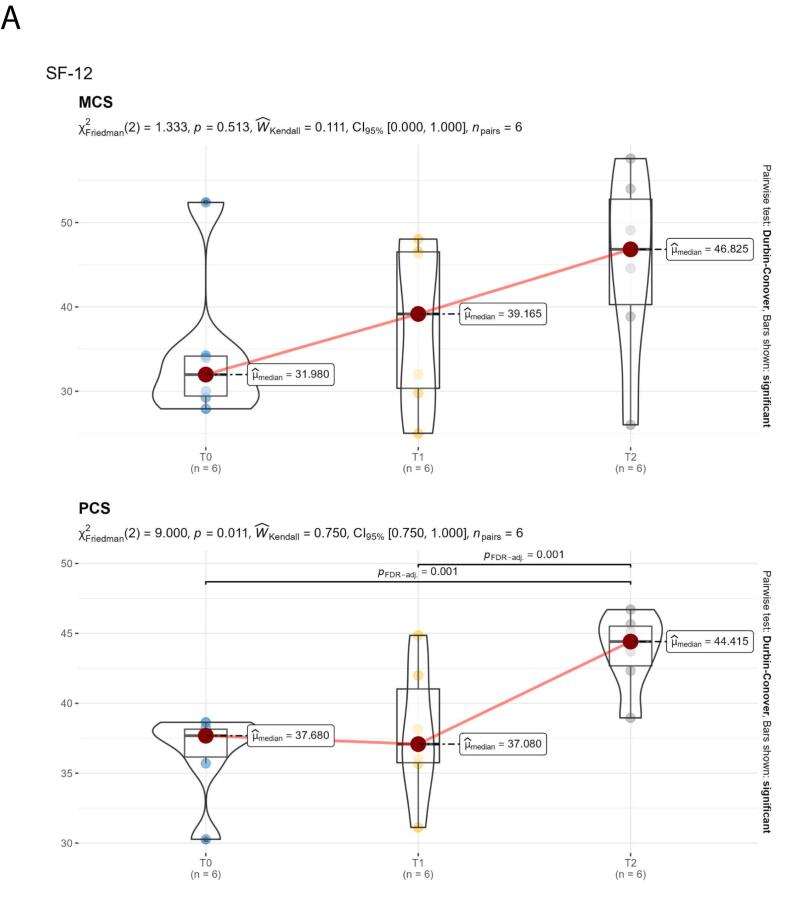

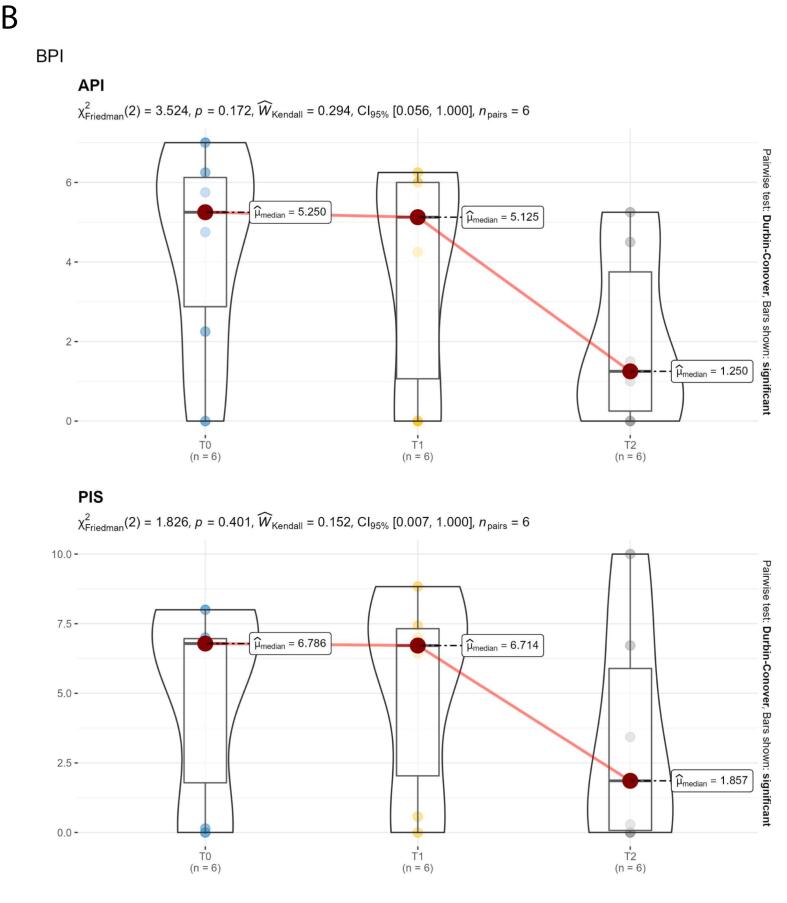

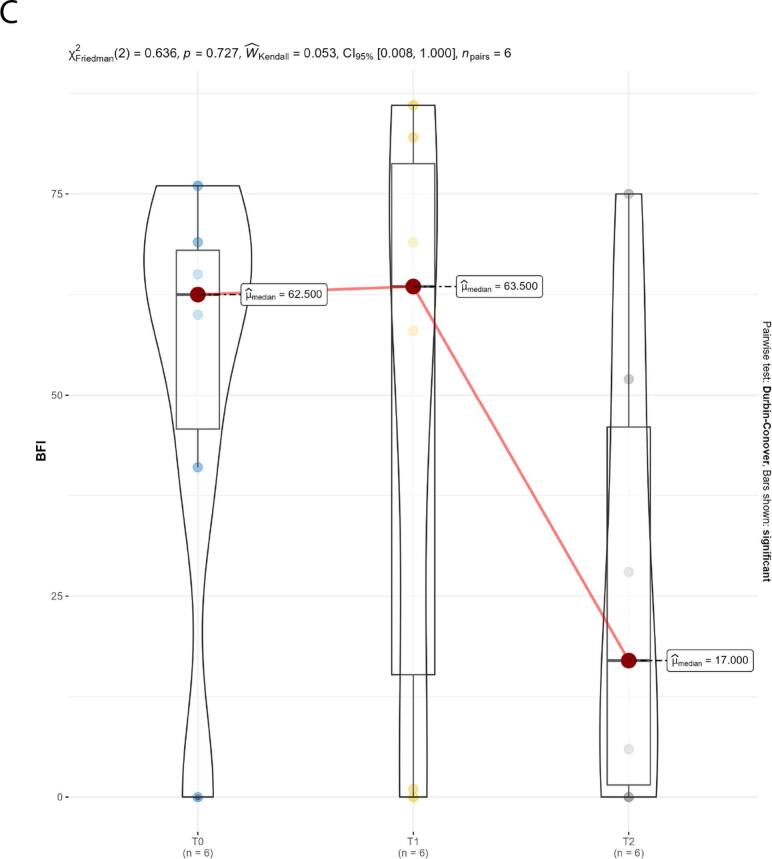
Quality of life. A. SF-12 dimensions (MCS and PCS) over time, reaveling improvement in both PCS and MCS, which was statically significant for the PCS component. B. Trends over time in the pain dimensions of the BPI. The two dimensions (API and PIS) report similar trends, revealing a decrease in the median value after 6 months. C. 12-month trend in BFI demonstrating a trend for reduction in the median value between 6 and 12 months of follow-up.

Data from six patients were available for the quality-of-life assessment. The median MCS and PCS scores of the SF-12v1 questionnaire showed an improvement over time; this improvement was statistically significant for the PCS component after the 6-month follow-up (*p* = 0.001; Fig. 1A). Median scores from the BPI questionnaire showed a trend of improvement after the 6-month follow-up, as well as the median BFI score (Fig. 1B, C).

## 4 Discussion

AIP is a severe multiorgan dysfunction disorder that can be fatal if not treated promptly [5]. At the same time, the chronic symptoms and the high disease burden affect the physical, emotional and social well-being of patients.

Recently, a new treatment modality involving small interfering RNA (siRNA) molecules, givosiran, was approved for the treatment of adults and adolescents with AIP aged >12 years, based on the positive results of the phase III trial ENVISION [2,9,19]. Results from the ENVISION study showed that long-term monthly treatment with givosiran leads to continuous and sustained reductions in annualized attack rate and in the use of hemin over time in patients with AHP, as well as improved quality of life, with an acceptable safety profile [2,9,19]. With regard to the real world, evidence on the effectiveness and safety of givosiran, especially in the long term, is still scant [[11], [18]].

Our case series involved 11 AIP patients (63% with a genetic diagnosis), with a mean age of 37 years at disease onset. The mean duration of givosiran treatment was 16 (±5) months.

In line with the literature data, our results show the effectiveness of givosiran in the reduction of the annualized attack rate, which in our series was reduced to zero in all patients after the start of the treatment. In addition, long-term givosiran treatment led to a sustained lowering of urinary ALA and PBG to normal levels, with a > 50% reduction of both parameters in about 60% of patients. The safety profile of givosiran observed in our case series was consistent with the previous analyses of ENVISION [16,19], with no additional emerging safety concerns observed.

Before the switch to givosiran, all patients included in our series were treated with glucose and/or heme preparations to manage acute attacks. However, after 3–4 weeks from the treatment, the symptoms recurred increasingly, requiring the use of hemin as a prophylactic treatment, with concomitant hospitalization whenever the disabling painful symptoms recurred, especially if associated with neurological symptoms and psychiatric disorders. According to the patients' report, full post-hemin well-being lasted about ten days. The introduction of givosiran therapy has, therefore, been a turning point for patients' quality of life, thanks to full well-being throughout the period between administrations. Moreover, it is worth mentioning that in our series, the effectiveness of givosiran was reported also with regard to the management of chronic symptoms, sustaining the value of this therapeutic approach in patients with a reduced annualized attack rate. This evidence is supported by the improvement in physical and mental health after 12 months of treatment, as assessed by the SF-12 physical and mental component summary (PCS and MCS), as well as by a trend for improvement in pain and fatigue. For completeness of information, it is right to emphasize that the perceived response to treatment was not the same for all patients. For example, in our series, we can distinguish patients who report perceiving a drastic improvement in their condition immediately after the start of givosiran treatment (e.g., patient 9 reported the complete resolution of symptoms after the first month of treatment, in parallel with the reduction in PBG values compared to baseline) and patients who report frequent abdominal pain symptoms associated with paresthesia during the first months of treatment, despite an improvement in ALA and PBG values, with resolution of symptoms after 5 months of treatment (e.g., patient 7).

Our study is limited by the small number of patients, as expected for a rare disease. However, our data contribute to the expansion of knowledge on long-term effects and effectiveness of givosiran treatment in real-world practice.

## 5 Conclusion

Our real-life experience supports the clinical evidence that long-term treatment with givosiran is well tolerated and able to provide sustained and continuous benefit to patients with AIP, as reflected by the reduction in frequency of attacks, hemin use and levels of ALA and PBG. Givosiran treatment was also associated with improvement in assessments of QoL, pain and fatigue.

## Funding

Editorial assistance was supported by Alnylam Pharmaceuticals for the medical writing and/or publication costs of this article, via a publications support request made by Claudio Carmine Guida that was granted to Polistudium SRL.

## Ethics approval

This retrospective review of patient data did not require ethical approval in accordance with local/national guidelines.

## Consent to participate

All the participants signed an informed consent form.

## CRediT authorship contribution statement

**Claudio Carmine Guida:** Writing – review & editing, Writing – original draft, Formal analysis, Data curation, Conceptualization. **Maria Nardella:** Writing – review & editing. **Aurora del Mar YS Perez:** Writing – review & editing, Formal analysis, Data curation. **Maria Savino:** Writing – review & editing, Formal analysis, Data curation. **Gaetano Ferrara:** Writing – review & editing, Formal analysis, Data curation. **Francesco Napolitano:** Writing – review & editing, Formal analysis, Data curation. **Annalisa Crisetti:** Writing – review & editing, Formal analysis, Data curation. **Francesco Aucella:** Writing – review & editing, Formal analysis, Data curation. **Filippo Aucella:** Writing – review & editing, Formal analysis, Data curation.

## Declaration of competing interest

None.

## Data Availability

The datasets generated during and/or analyzed during the current study are available from the corresponding author upon reasonable request.
